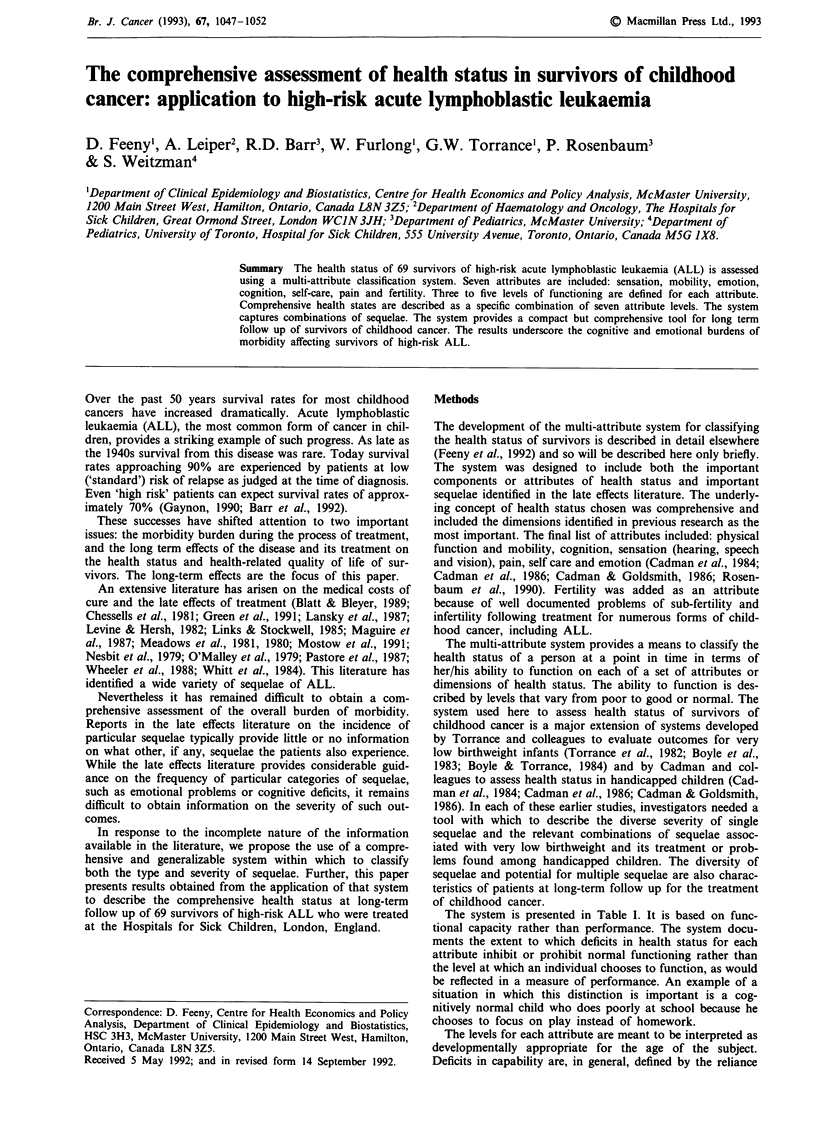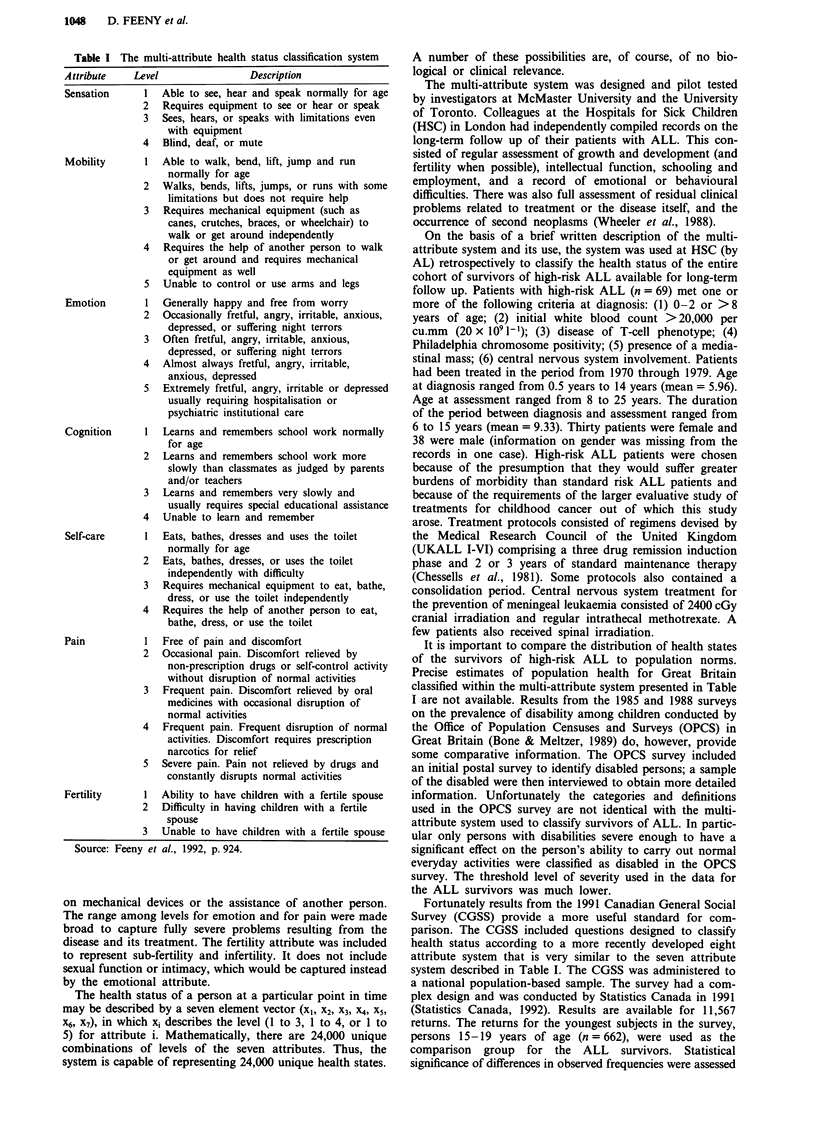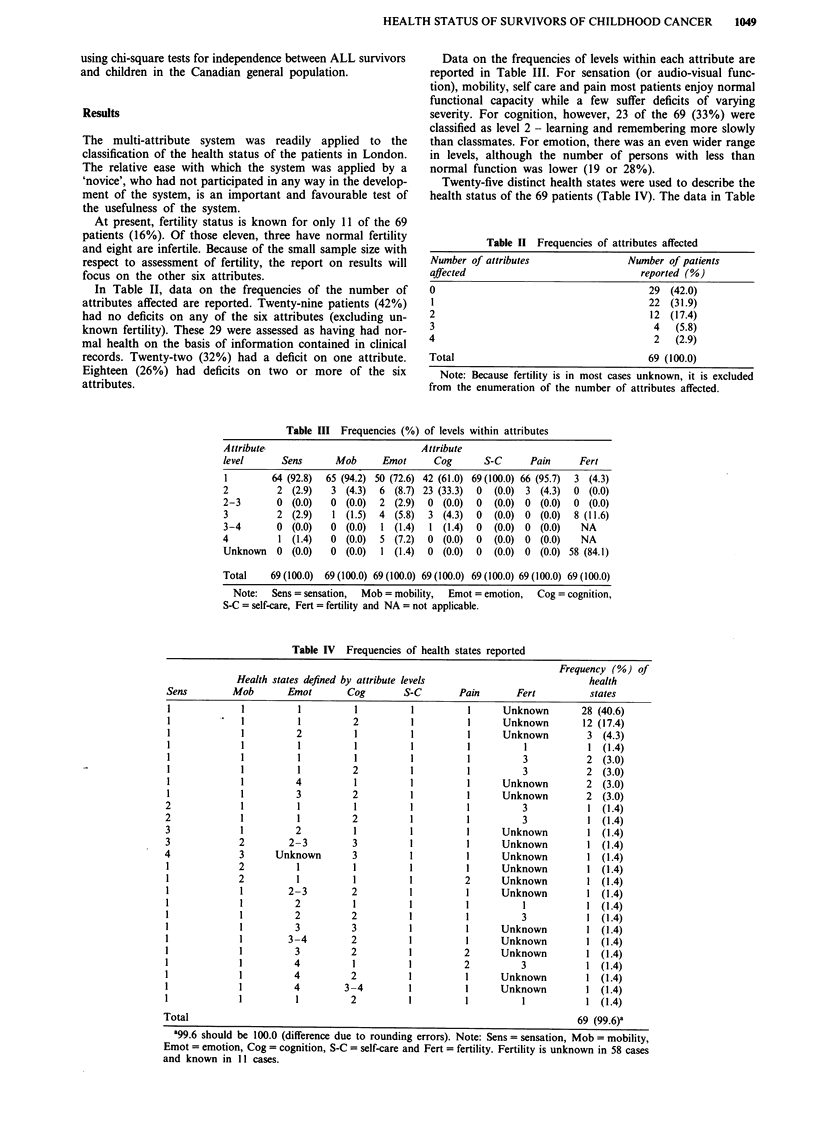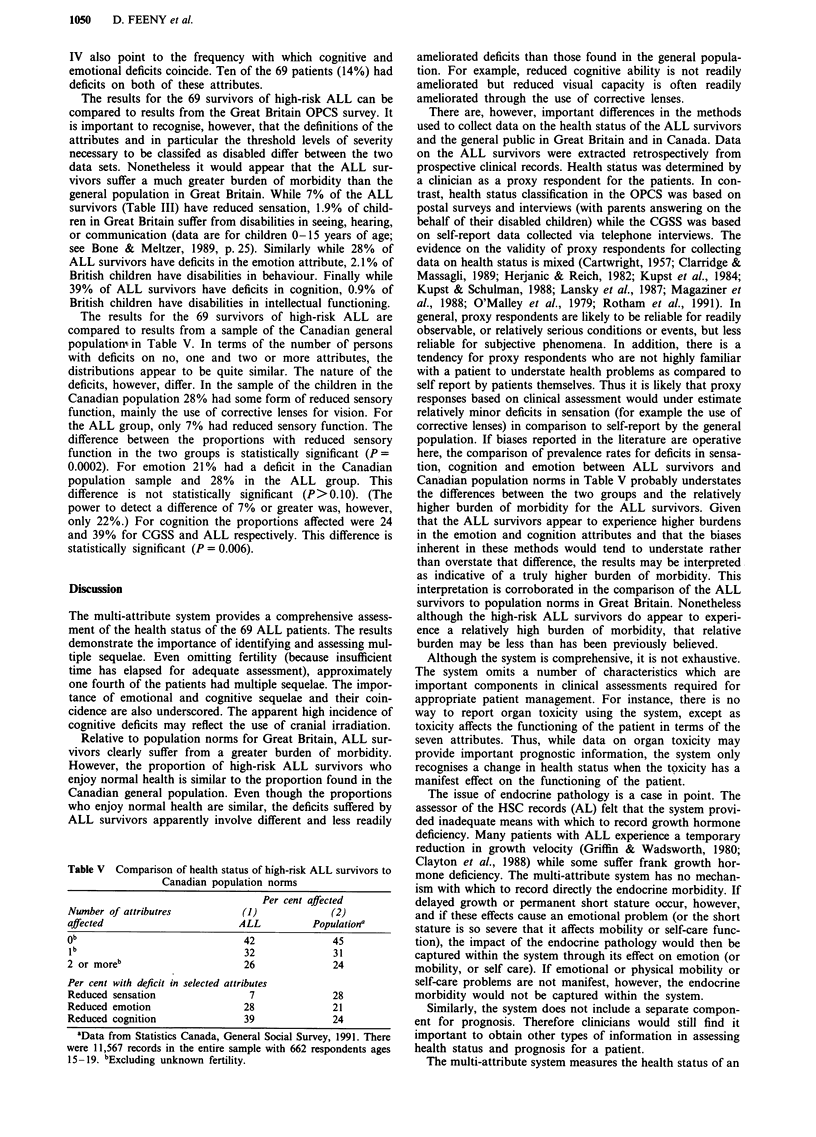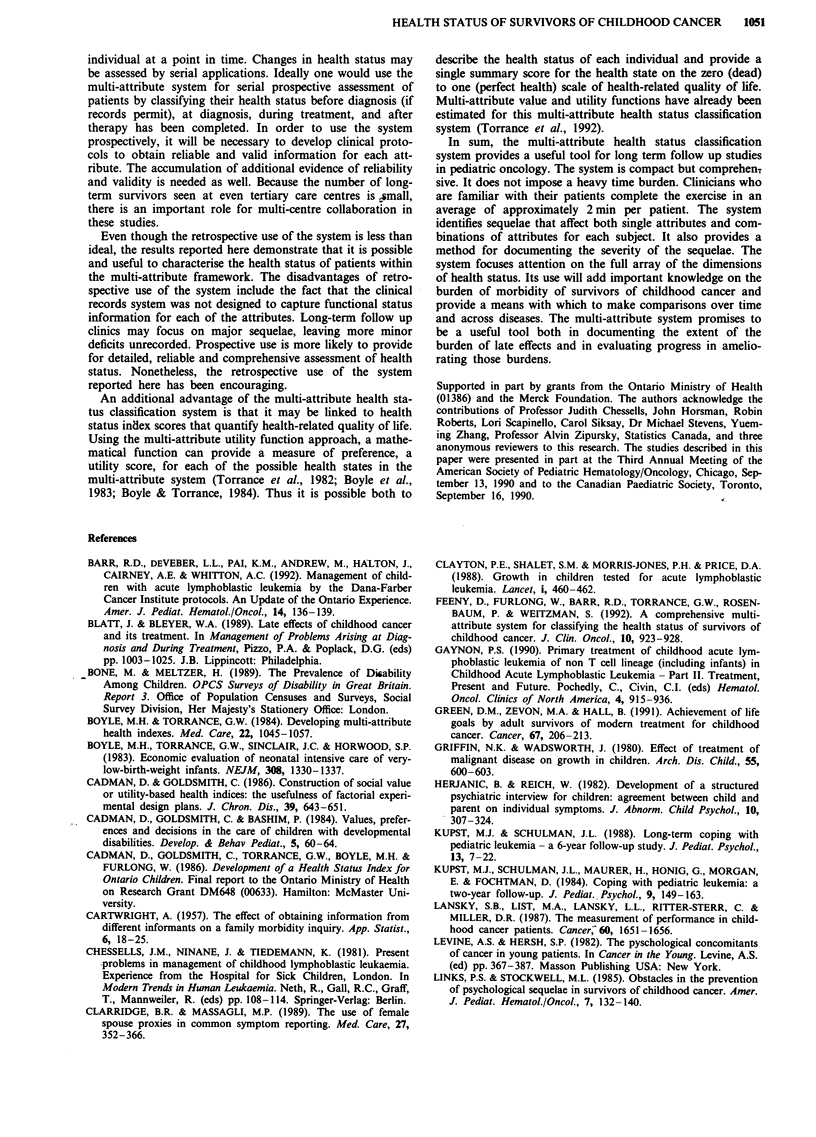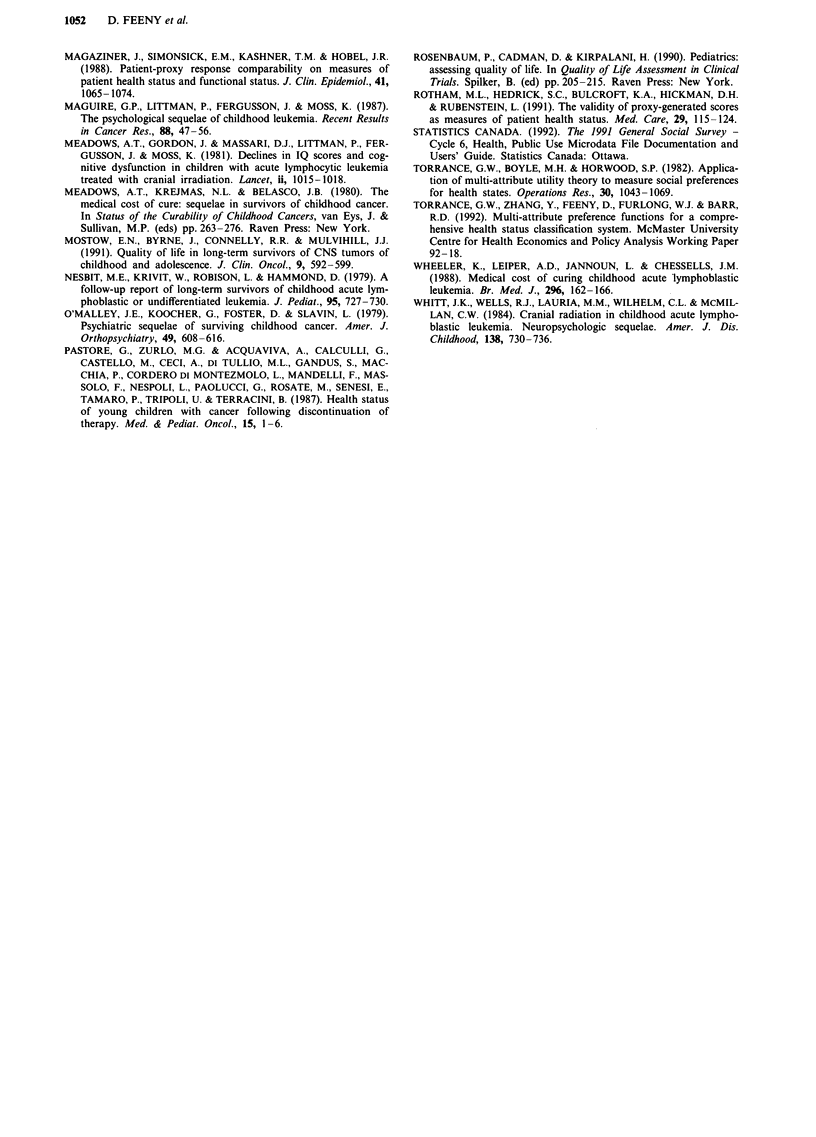# The comprehensive assessment of health status in survivors of childhood cancer: application to high-risk acute lymphoblastic leukaemia.

**DOI:** 10.1038/bjc.1993.192

**Published:** 1993-05

**Authors:** D. Feeny, A. Leiper, R. D. Barr, W. Furlong, G. W. Torrance, P. Rosenbaum, S. Weitzman

**Affiliations:** Department of Clinical Epidemiology and Biostatistics, McMaster University, Ontario, Canada.

## Abstract

The health status of 69 survivors of high-risk acute lymphoblastic leukaemia (ALL) is assessed using a multi-attribute classification system. Seven attributes are included: sensation, mobility, emotion, cognition, self-care, pain and fertility. Three to five levels of functioning are defined for each attribute. Comprehensive health states are described as a specific combination of seven attribute levels. The system captures combinations of sequelae. The system provides a compact but comprehensive tool for long term follow up of survivors of childhood cancer. The results underscore the cognitive and emotional burdens of morbidity affecting survivors of high-risk ALL.


Br. J. Cancer (1993), 67, 1047–1052 © Macmillan Press Ltd., 1993

### The comprehensive assessment of health status in survivors of childhood cancer: application to high-risk acute lymphoblastic leukaemia

D. Feeny[1], A. Leiper[2], R.D. Barr[3], W. Furlong[1], G.W. Torrance[1], P. Rosenbaum[3] & S. Weitzman[4]

[1]*Department of Clinical Epidemiology and Biostatistics, Centre for Health Economics and Policy Analysis, McMaster University, 1200 Main Street West, Hamilton, Ontario, Canada L8N 3Z5;* [2]*Department of Haematology and Oncology, The Hospitals for Sick Children, Great Ormond Street, London WC1N 3JH;* [3]*Department of Pediatrics, McMaster University;* [4]*Department of Pediatrics, University of Toronto, Hospital for Sick Children, 555 University Avenue, Toronto, Ontario, Canada M5G 1X8.*

**Summary** The health status of 69 survivors of high-risk acute lymphoblastic leukaemia (ALL) is assessed using a multi-attribute classification system. Seven attributes are included: sensation, mobility, emotion, cognition, self-care, pain and fertility. Three to five levels of functioning are defined for each attribute. Comprehensive health states are described as a specific combination of seven attribute levels. The system captures combinations of sequelae. The system provides a compact but comprehensive tool for long term follow up of survivors of childhood cancer. The results underscore the cognitive and emotional burdens of morbidity affecting survivors of high-risk ALL.

Over the past 50 years survival rates for most childhood cancers have increased dramatically. Acute lymphoblastic leukaemia (ALL), the most common form of cancer in children, provides a striking example of such progress. As late as the 1940s survival from this disease was rare. Today survival rates approaching 90% are experienced by patients at low ('standard') risk of relapse as judged at the time of diagnosis. Even 'high risk' patients can expect survival rates of approximately 70% (Gaynon, 1990; Barr *et al.*, 1992).

These successes have shifted attention to two important issues: the morbidity burden during the process of treatment, and the long term effects of the disease and its treatment on the health status and health-related quality of life of survivors. The long-term effects are the focus of this paper.

An extensive literature has arisen on the medical costs of cure and the late effects of treatment (Blatt & Bleyer, 1989; Chessells *et al.*, 1981; Green *et al.*, 1991; Lansky *et al.*, 1987; Levine & Hersh, 1982; Links & Stockwell, 1985; Maguire *et al.*, 1987; Meadows *et al.*, 1981, 1980; Mostow *et al.*, 1991; Nesbit *et al.*, 1979; O'Malley *et al.*, 1979; Pastore *et al.*, 1987; Wheeler *et al.*, 1988; Whitt *et al.*, 1984). This literature has identified a wide variety of sequelae of ALL.

Nevertheless it has remained difficult to obtain a comprehensive assessment of the overall burden of morbidity. Reports in the late effects literature on the incidence of particular sequelae typically provide little or no information on what other, if any, sequelae the patients also experience. While the late effects literature provides considerable guidance on the frequency of particular categories of sequelae, such as emotional problems or cognitive deficits, it remains difficult to obtain information on the severity of such outcomes.

In response to the incomplete nature of the information available in the literature, we propose the use of a comprehensive and generalizable system within which to classify both the type and severity of sequelae. Further, this paper presents results obtained from the application of that system to describe the comprehensive health status at long-term follow up of 69 survivors of high-risk ALL who were treated at the Hospitals for Sick Children, London, England.

Correspondence: D. Feeny, Centre for Health Economics and Policy Analysis, Department of Clinical Epidemiology and Biostatistics, HSC 3H3, McMaster University, 1200 Main Street West, Hamilton, Ontario, Canada L8N 3Z5.

Received 5 May 1992; and in revised form 14 September 1992.

### Methods

The development of the multi-attribute system for classifying the health status of survivors is described in detail elsewhere (Feeny *et al.*, 1992) and so will be described here only briefly. The system was designed to include both the important components or attributes of health status and important sequelae identified in the late effects literature. The underlying concept of health status chosen was comprehensive and included the dimensions identified in previous research as the most important. The final list of attributes included: physical function and mobility, cognition, sensation (hearing, speech and vision), pain, self care and emotion (Cadman *et al.*, 1984; Cadman *et al.*, 1986; Cadman & Goldsmith, 1986; Rosenbaum *et al.*, 1990). Fertility was added as an attribute because of well documented problems of sub-fertility and infertility following treatment for numerous forms of childhood cancer, including ALL.

The multi-attribute system provides a means to classify the health status of a person at a point in time in terms of her/his ability to function on each of a set of attributes or dimensions of health status. The ability to function is described by levels that vary from poor to good or normal. The system used here to assess health status of survivors of childhood cancer is a major extension of systems developed by Torrance and colleagues to evaluate outcomes for very low birthweight infants (Torrance *et al.*, 1982; Boyle *et al.*, 1983; Boyle & Torrance, 1984) and by Cadman and colleagues to assess health status in handicapped children (Cadman *et al.*, 1984; Cadman *et al.*, 1986; Cadman & Goldsmith, 1986). In each of these earlier studies, investigators needed a tool with which to describe the diverse severity of single sequelae and the relevant combinations of sequelae associated with very low birthweight and its treatment or problems found among handicapped children. The diversity of sequelae and potential for multiple sequelae are also characteristics of patients at long-term follow up for the treatment of childhood cancer.

The system is presented in Table I. It is based on functional capacity rather than performance. The system documents the extent to which deficits in health status for each attribute inhibit or prohibit normal functioning rather than the level at which an individual chooses to function, as would be reflected in a measure of performance. An example of a situation in which this distinction is important is a cognitively normal child who does poorly at school because he chooses to focus on play instead of homework.

The levels for each attribute are meant to be interpreted as developmentally appropriate for the age of the subject. Deficits in capability are, in general, defined by the reliance

**Table I** The multi-attribute health status classification system

| *Attribute* | *Level* | *Description* |
|---|---|---|
| Sensation | 1 | Able to see, hear and speak normally for age |
| | 2 | Requires equipment to see or hear or speak |
| | 3 | Sees, hears, or speaks with limitations even with equipment |
| | 4 | Blind, deaf, or mute |
| Mobility | 1 | Able to walk, bend, lift, jump and run normally for age |
| | 2 | Walks, bends, lifts, jumps, or runs with some limitations but does not require help |
| | 3 | Requires mechanical equipment (such as canes, crutches, braces, or wheelchair) to walk or get around independently |
| | 4 | Requires the help of another person to walk or get around and requires mechanical equipment as well |
| | 5 | Unable to control or use arms and legs |
| Emotion | 1 | Generally happy and free from worry |
| | 2 | Occasionally fretful, angry, irritable, anxious, depressed, or suffering night terrors |
| | 3 | Often fretful, angry, irritable, anxious, depressed, or suffering night terrors |
| | 4 | Almost always fretful, angry, irritable, anxious, depressed |
| | 5 | Extremely fretful, angry, irritable or depressed usually requiring hospitalisation or psychiatric institutional care |
| Cognition | 1 | Learns and remembers school work normally for age |
| | 2 | Learns and remembers school work more slowly than classmates as judged by parents and/or teachers |
| | 3 | Learns and remembers very slowly and usually requires special educational assistance |
| | 4 | Unable to learn and remember |
| Self-care | 1 | Eats, bathes, dresses and uses the toilet normally for age |
| | 2 | Eats, bathes, dresses, or uses the toilet independently with difficulty |
| | 3 | Requires mechanical equipment to eat, bathe, dress, or use the toilet independently |
| | 4 | Requires the help of another person to eat, bathe, dress, or use the toilet |
| Pain | 1 | Free of pain and discomfort |
| | 2 | Occasional pain. Discomfort relieved by non-prescription drugs or self-control activity without disruption of normal activities |
| | 3 | Frequent pain. Discomfort relieved by oral medicines with occasional disruption of normal activities |
| | 4 | Frequent pain. Frequent disruption of normal activities. Discomfort requires prescription narcotics for relief |
| | 5 | Severe pain. Pain not relieved by drugs and constantly disrupts normal activities |
| Fertility | 1 | Ability to have children with a fertile spouse |
| | 2 | Difficulty in having children with a fertile spouse |
| | 3 | Unable to have children with a fertile spouse |

Source: Feeny *et al.*, 1992, p. 924.

on mechanical devices or the assistance of another person. The range among levels for emotion and for pain were made broad to capture fully severe problems resulting from the disease and its treatment. The fertility attribute was included to represent sub-fertility and infertility. It does not include sexual function or intimacy, which would be captured instead by the emotional attribute.

The health status of a person at a particular point in time may be described by a seven element vector ($x_1$, $x_2$, $x_3$, $x_4$, $x_5$, $x_6$, $x_7$), in which $x_i$ describes the level (1 to 3, 1 to 4, or 1 to 5) for attribute i. Mathematically, there are 24,000 unique combinations of levels of the seven attributes. Thus, the system is capable of representing 24,000 unique health states. A number of these possibilities are, of course, of no biological or clinical relevance.

The multi-attribute system was designed and pilot tested by investigators at McMaster University and the University of Toronto. Colleagues at the Hospitals for Sick Children (HSC) in London had independently compiled records on the long-term follow up of their patients with ALL. This consisted of regular assessment of growth and development (and fertility when possible), intellectual function, schooling and employment, and a record of emotional or behavioural difficulties. There was also full assessment of residual clinical problems related to treatment or the disease itself, and the occurrence of second neoplasms (Wheeler *et al.*, 1988).

On the basis of a brief written description of the multi-attribute system and its use, the system was used at HSC (by AL) retrospectively to classify the health status of the entire cohort of survivors of high-risk ALL available for long-term follow up. Patients with high-risk ALL ($n = 69$) met one or more of the following criteria at diagnosis: (1) 0–2 or $>8$ years of age; (2) initial white blood count $>20{,}000$ per cu.mm ($20 \times 10^9 l^{-1}$); (3) disease of T-cell phenotype; (4) Philadelphia chromosome positivity; (5) presence of a mediastinal mass; (6) central nervous system involvement. Patients had been treated in the period from 1970 through 1979. Age at diagnosis ranged from 0.5 years to 14 years (mean = 5.96). Age at assessment ranged from 8 to 25 years. The duration of the period between diagnosis and assessment ranged from 6 to 15 years (mean = 9.33). Thirty patients were female and 38 were male (information on gender was missing from the records in one case). High-risk ALL patients were chosen because of the presumption that they would suffer greater burdens of morbidity than standard risk ALL patients and because of the requirements of the larger evaluative study of treatments for childhood cancer out of which this study arose. Treatment protocols consisted of regimens devised by the Medical Research Council of the United Kingdom (UKALL I-VI) comprising a three drug remission induction phase and 2 or 3 years of standard maintenance therapy (Chessells *et al.*, 1981). Some protocols also contained a consolidation period. Central nervous system treatment for the prevention of meningeal leukaemia consisted of 2400 cGy cranial irradiation and regular intrathecal methotrexate. A few patients also received spinal irradiation.

It is important to compare the distribution of health states of the survivors of high-risk ALL to population norms. Precise estimates of population health for Great Britain classified within the multi-attribute system presented in Table I are not available. Results from the 1985 and 1988 surveys on the prevalence of disability among children conducted by the Office of Population Censuses and Surveys (OPCS) in Great Britain (Bone & Meltzer, 1989) do, however, provide some comparative information. The OPCS survey included an initial postal survey to identify disabled persons; a sample of the disabled were then interviewed to obtain more detailed information. Unfortunately the categories and definitions used in the OPCS survey are not identical with the multi-attribute system used to classify survivors of ALL. In particular only persons with disabilities severe enough to have a significant effect on the person's ability to carry out normal everyday activities were classified as disabled in the OPCS survey. The threshold level of severity used in the data for the ALL survivors was much lower.

Fortunately results from the 1991 Canadian General Social Survey (CGSS) provide a more useful standard for comparison. The CGSS included questions designed to classify health status according to a more recently developed eight attribute system that is very similar to the seven attribute system described in Table I. The CGSS was administered to a national population-based sample. The survey had a complex design and was conducted by Statistics Canada in 1991 (Statistics Canada, 1992). Results are available for 11,567 returns. The returns for the youngest subjects in the survey, persons 15–19 years of age ($n = 662$), were used as the comparison group for the ALL survivors. Statistical significance of differences in observed frequencies were assessed using chi-square tests for independence between ALL survivors and children in the Canadian general population.

### Results

The multi-attribute system was readily applied to the classification of the health status of the patients in London. The relative ease with which the system was applied by a 'novice', who had not participated in any way in the development of the system, is an important and favourable test of the usefulness of the system.

At present, fertility status is known for only 11 of the 69 patients (16%). Of those eleven, three have normal fertility and eight are infertile. Because of the small sample size with respect to assessment of fertility, the report on results will focus on the other six attributes.

In Table II, data on the frequencies of the number of attributes affected are reported. Twenty-nine patients (42%) had no deficits on any of the six attributes (excluding unknown fertility). These 29 were assessed as having had normal health on the basis of information contained in clinical records. Twenty-two (32%) had a deficit on one attribute. Eighteen (26%) had deficits on two or more of the six attributes.

Data on the frequencies of levels within each attribute are reported in Table III. For sensation (or audio-visual function), mobility, self care and pain most patients enjoy normal functional capacity while a few suffer deficits of varying severity. For cognition, however, 23 of the 69 (33%) were classified as level 2 – learning and remembering more slowly than classmates. For emotion, there was an even wider range in levels, although the number of persons with less than normal function was lower (19 or 28%).

Twenty-five distinct health states were used to describe the health status of the 69 patients (Table IV). The data in Table

**Table II** Frequencies of attributes affected

| *Number of attributes affected* | *Number of patients reported (%)* |
|---|---|
| 0 | 29 (42.0) |
| 1 | 22 (31.9) |
| 2 | 12 (17.4) |
| 3 | 4 (5.8) |
| 4 | 2 (2.9) |
| Total | 69 (100.0) |

Note: Because fertility is in most cases unknown, it is excluded from the enumeration of the number of attributes affected.

**Table III** Frequencies (%) of levels within attributes

| *Attribute level* | *Sens* | *Mob* | *Emot* | *Cog* | *S-C* | *Pain* | *Fert* |
|---|---|---|---|---|---|---|---|
| 1 | 64 (92.8) | 65 (94.2) | 50 (72.6) | 42 (61.0) | 69 (100.0) | 66 (95.7) | 3 (4.3) |
| 2 | 2 (2.9) | 3 (4.3) | 6 (8.7) | 23 (33.3) | 0 (0.0) | 3 (4.3) | 0 (0.0) |
| 2–3 | 0 (0.0) | 0 (0.0) | 2 (2.9) | 0 (0.0) | 0 (0.0) | 0 (0.0) | 0 (0.0) |
| 3 | 2 (2.9) | 1 (1.5) | 4 (5.8) | 3 (4.3) | 0 (0.0) | 0 (0.0) | 8 (11.6) |
| 3–4 | 0 (0.0) | 0 (0.0) | 1 (1.4) | 1 (1.4) | 0 (0.0) | 0 (0.0) | NA |
| 4 | 1 (1.4) | 0 (0.0) | 5 (7.2) | 0 (0.0) | 0 (0.0) | 0 (0.0) | NA |
| Unknown | 0 (0.0) | 0 (0.0) | 1 (1.4) | 0 (0.0) | 0 (0.0) | 0 (0.0) | 58 (84.1) |
| Total | 69 (100.0) | 69 (100.0) | 69 (100.0) | 69 (100.0) | 69 (100.0) | 69 (100.0) | 69 (100.0) |

Note: Sens = sensation, Mob = mobility, Emot = emotion, Cog = cognition, S-C = self-care, Fert = fertility and NA = not applicable.

**Table IV** Frequencies of health states reported

| *Sens* | *Mob* | *Emot* | *Cog* | *S-C* | *Pain* | *Fert* | *Frequency (%) of health states* |
|---|---|---|---|---|---|---|---|
| 1 | 1 | 1 | 1 | 1 | 1 | Unknown | 28 (40.6) |
| 1 | 1 | 1 | 2 | 1 | 1 | Unknown | 12 (17.4) |
| 1 | 1 | 2 | 1 | 1 | 1 | Unknown | 3 (4.3) |
| 1 | 1 | 1 | 1 | 1 | 1 | 1 | 1 (1.4) |
| 1 | 1 | 1 | 1 | 1 | 1 | 3 | 2 (3.0) |
| 1 | 1 | 1 | 2 | 1 | 1 | 3 | 2 (3.0) |
| 1 | 1 | 4 | 1 | 1 | 1 | Unknown | 2 (3.0) |
| 1 | 1 | 3 | 2 | 1 | 1 | Unknown | 2 (3.0) |
| 2 | 1 | 1 | 1 | 1 | 1 | 3 | 1 (1.4) |
| 2 | 1 | 1 | 2 | 1 | 1 | 3 | 1 (1.4) |
| 3 | 1 | 2 | 1 | 1 | 1 | Unknown | 1 (1.4) |
| 3 | 2 | 2–3 | 3 | 1 | 1 | Unknown | 1 (1.4) |
| 4 | 3 | Unknown | 3 | 1 | 1 | Unknown | 1 (1.4) |
| 1 | 2 | 1 | 1 | 1 | 1 | Unknown | 1 (1.4) |
| 1 | 2 | 1 | 1 | 1 | 2 | Unknown | 1 (1.4) |
| 1 | 1 | 2–3 | 2 | 1 | 1 | Unknown | 1 (1.4) |
| 1 | 1 | 2 | 1 | 1 | 1 | 1 | 1 (1.4) |
| 1 | 1 | 2 | 2 | 1 | 1 | 3 | 1 (1.4) |
| 1 | 1 | 3 | 3 | 1 | 1 | Unknown | 1 (1.4) |
| 1 | 1 | 3–4 | 2 | 1 | 1 | Unknown | 1 (1.4) |
| 1 | 1 | 3 | 2 | 1 | 2 | Unknown | 1 (1.4) |
| 1 | 1 | 4 | 1 | 1 | 2 | 3 | 1 (1.4) |
| 1 | 1 | 4 | 2 | 1 | 1 | Unknown | 1 (1.4) |
| 1 | 1 | 4 | 3–4 | 1 | 1 | Unknown | 1 (1.4) |
| 1 | 1 | 1 | 2 | 1 | 1 | 1 | 1 (1.4) |
| Total | | | | | | | 69 (99.6)[a] |

[a]99.6 should be 100.0 (difference due to rounding errors). Note: Sens = sensation, Mob = mobility, Emot = emotion, Cog = cognition, S-C = self-care and Fert = fertility. Fertility is unknown in 58 cases and known in 11 cases.

IV also point to the frequency with which cognitive and emotional deficits coincide. Ten of the 69 patients (14%) had deficits on both of these attributes.

The results for the 69 survivors of high-risk ALL can be compared to results from the Great Britain OPCS survey. It is important to recognise, however, that the definitions of the attributes and in particular the threshold levels of severity necessary to be classifed as disabled differ between the two data sets. Nonetheless it would appear that the ALL survivors suffer a much greater burden of morbidity than the general population in Great Britain. While 7% of the ALL survivors (Table III) have reduced sensation, 1.9% of children in Great Britain suffer from disabilities in seeing, hearing, or communication (data are for children 0–15 years of age; see Bone & Meltzer, 1989, p. 25). Similarly while 28% of ALL survivors have deficits in the emotion attribute, 2.1% of British children have disabilities in behaviour. Finally while 39% of ALL survivors have deficits in cognition, 0.9% of British children have disabilities in intellectual functioning.

The results for the 69 survivors of high-risk ALL are compared to results from a sample of the Canadian general population in Table V. In terms of the number of persons with deficits on no, one and two or more attributes, the distributions appear to be quite similar. The nature of the deficits, however, differ. In the sample of the children in the Canadian population 28% had some form of reduced sensory function, mainly the use of corrective lenses for vision. For the ALL group, only 7% had reduced sensory function. The difference between the proportions with reduced sensory function in the two groups is statistically significant ($P = 0.0002$). For emotion 21% had a deficit in the Canadian population sample and 28% in the ALL group. This difference is not statistically significant ($P > 0.10$). (The power to detect a difference of 7% or greater was, however, only 22%.) For cognition the proportions affected were 24 and 39% for CGSS and ALL respectively. This difference is statistically significant ($P = 0.006$).

### Discussion

The multi-attribute system provides a comprehensive assessment of the health status of the 69 ALL patients. The results demonstrate the importance of identifying and assessing multiple sequelae. Even omitting fertility (because insufficient time has elapsed for adequate assessment), approximately one fourth of the patients had multiple sequelae. The importance of emotional and cognitive sequelae and their coincidence are also underscored. The apparent high incidence of cognitive deficits may reflect the use of cranial irradiation.

Relative to population norms for Great Britain, ALL survivors clearly suffer from a greater burden of morbidity. However, the proportion of high-risk ALL survivors who enjoy normal health is similar to the proportion found in the Canadian general population. Even though the proportions who enjoy normal health are similar, the deficits suffered by ALL survivors apparently involve different and less readily

**Table V** Comparison of health status of high-risk ALL survivors to Canadian population norms

| Number of attributres affected | Per cent affected (1) ALL | (2) Population[a] |
|---|---|---|
| 0[b] | 42 | 45 |
| 1[b] | 32 | 31 |
| 2 or more[b] | 26 | 24 |
| *Per cent with deficit in selected attributes* | | |
| Reduced sensation | 7 | 28 |
| Reduced emotion | 28 | 21 |
| Reduced cognition | 39 | 24 |

[a]Data from Statistics Canada, General Social Survey, 1991. There were 11,567 records in the entire sample with 662 respondents ages 15–19. [b]Excluding unknown fertility.

ameliorated deficits than those found in the general population. For example, reduced cognitive ability is not readily ameliorated but reduced visual capacity is often readily ameliorated through the use of corrective lenses.

There are, however, important differences in the methods used to collect data on the health status of the ALL survivors and the general public in Great Britain and in Canada. Data on the ALL survivors were extracted retrospectively from prospective clinical records. Health status was determined by a clinician as a proxy respondent for the patients. In contrast, health status classification in the OPCS was based on postal surveys and interviews (with parents answering on the behalf of their disabled children) while the CGSS was based on self-report data collected via telephone interviews. The evidence on the validity of proxy respondents for collecting data on health status is mixed (Cartwright, 1957; Clarridge & Massagli, 1989; Herjanic & Reich, 1982; Kupst et al., 1984; Kupst & Schulman, 1988; Lansky et al., 1987; Magaziner et al., 1988; O'Malley et al., 1979; Rotham et al., 1991). In general, proxy respondents are likely to be reliable for readily observable, or relatively serious conditions or events, but less reliable for subjective phenomena. In addition, there is a tendency for proxy respondents who are not highly familiar with a patient to understate health problems as compared to self report by patients themselves. Thus it is likely that proxy responses based on clinical assessment would under estimate relatively minor deficits in sensation (for example the use of corrective lenses) in comparison to self-report by the general population. If biases reported in the literature are operative here, the comparison of prevalence rates for deficits in sensation, cognition and emotion between ALL survivors and Canadian population norms in Table V probably understates the differences between the two groups and the relatively higher burden of morbidity for the ALL survivors. Given that the ALL survivors appear to experience higher burdens in the emotion and cognition attributes and that the biases inherent in these methods would tend to understate rather than overstate that difference, the results may be interpreted as indicative of a truly higher burden of morbidity. This interpretation is corroborated in the comparison of the ALL survivors to population norms in Great Britain. Nonetheless although the high-risk ALL survivors do appear to experience a relatively high burden of morbidity, that relative burden may be less than has been previously believed.

Although the system is comprehensive, it is not exhaustive. The system omits a number of characteristics which are important components in clinical assessments required for appropriate patient management. For instance, there is no way to report organ toxicity using the system, except as toxicity affects the functioning of the patient in terms of the seven attributes. Thus, while data on organ toxicity may provide important prognostic information, the system only recognises a change in health status when the toxicity has a manifest effect on the functioning of the patient.

The issue of endocrine pathology is a case in point. The assessor of the HSC records (AL) felt that the system provided inadequate means with which to record growth hormone deficiency. Many patients with ALL experience a temporary reduction in growth velocity (Griffin & Wadsworth, 1980; Clayton et al., 1988) while some suffer frank growth hormone deficiency. The multi-attribute system has no mechanism with which to record directly the endocrine morbidity. If delayed growth or permanent short stature occur, however, and if these effects cause an emotional problem (or the short stature is so severe that it affects mobility or self-care function), the impact of the endocrine pathology would then be captured within the system through its effect on emotion (or mobility, or self care). If emotional or physical mobility or self-care problems are not manifest, however, the endocrine morbidity would not be captured within the system.

Similarly, the system does not include a separate component for prognosis. Therefore clinicians would still find it important to obtain other types of information in assessing health status and prognosis for a patient.

The multi-attribute system measures the health status of an individual at a point in time. Changes in health status may be assessed by serial applications. Ideally one would use the multi-attribute system for serial prospective assessment of patients by classifying their health status before diagnosis (if records permit), at diagnosis, during treatment, and after therapy has been completed. In order to use the system prospectively, it will be necessary to develop clinical protocols to obtain reliable and valid information for each attribute. The accumulation of additional evidence of reliability and validity is needed as well. Because the number of long-term survivors seen at even tertiary care centres is small, there is an important role for multi-centre collaboration in these studies.

Even though the retrospective use of the system is less than ideal, the results reported here demonstrate that it is possible and useful to characterise the health status of patients within the multi-attribute framework. The disadvantages of retrospective use of the system include the fact that the clinical records system was not designed to capture functional status information for each of the attributes. Long-term follow up clinics may focus on major sequelae, leaving more minor deficits unrecorded. Prospective use is more likely to provide for detailed, reliable and comprehensive assessment of health status. Nonetheless, the retrospective use of the system reported here has been encouraging.

An additional advantage of the multi-attribute health status classification system is that it may be linked to health status index scores that quantify health-related quality of life. Using the multi-attribute utility function approach, a mathematical function can provide a measure of preference, a utility score, for each of the possible health states in the multi-attribute system (Torrance *et al.*, 1982; Boyle *et al.*, 1983; Boyle & Torrance, 1984). Thus it is possible both to describe the health status of each individual and provide a single summary score for the health state on the zero (dead) to one (perfect health) scale of health-related quality of life. Multi-attribute value and utility functions have already been estimated for this multi-attribute health status classification system (Torrance *et al.*, 1992).

In sum, the multi-attribute health status classification system provides a useful tool for long term follow up studies in pediatric oncology. The system is compact but comprehensive. It does not impose a heavy time burden. Clinicians who are familiar with their patients complete the exercise in an average of approximately 2 min per patient. The system identifies sequelae that affect both single attributes and combinations of attributes for each subject. It also provides a method for documenting the severity of the sequelae. The system focuses attention on the full array of the dimensions of health status. Its use will add important knowledge on the burden of morbidity of survivors of childhood cancer and provide a means with which to make comparisons over time and across diseases. The multi-attribute system promises to be a useful tool both in documenting the extent of the burden of late effects and in evaluating progress in ameliorating those burdens.

Supported in part by grants from the Ontario Ministry of Health (01386) and the Merck Foundation. The authors acknowledge the contributions of Professor Judith Chessells, John Horsman, Robin Roberts, Lori Scapinello, Carol Siksay, Dr Michael Stevens, Yueming Zhang, Professor Alvin Zipursky, Statistics Canada, and three anonymous reviewers to this research. The studies described in this paper were presented in part at the Third Annual Meeting of the American Society of Pediatric Hematology/Oncology, Chicago, September 13, 1990 and to the Canadian Paediatric Society, Toronto, September 16, 1990.

### References

BARR, R.D., DEVEBER, L.L., PAI, K.M., ANDREW, M., HALTON, J., CAIRNEY, A.E. & WHITTON, A.C. (1992). Management of children with acute lymphoblastic leukemia by the Dana-Farber Cancer Institute protocols. An Update of the Ontario Experience. *Amer. J. Pediat. Hematol./Oncol.*, **14**, 136–139.

BLATT, J. & BLEYER, W.A. (1989). Late effects of childhood cancer and its treatment. In *Management of Problems Arising at Diagnosis and During Treatment*, Pizzo, P.A. & Poplack, D.G. (eds) pp. 1003–1025. J.B. Lippincott: Philadelphia.

BONE, M. & MELTZER, H. (1989). The Prevalence of Disability Among Children. *OPCS Surveys of Disability in Great Britain. Report 3*. Office of Population Censuses and Surveys, Social Survey Division, Her Majesty's Stationery Office: London.

BOYLE, M.H. & TORRANCE, G.W. (1984). Developing multi-attribute health indexes. *Med. Care*, **22**, 1045–1057.

BOYLE, M.H., TORRANCE, G.W., SINCLAIR, J.C. & HORWOOD, S.P. (1983). Economic evaluation of neonatal intensive care of very-low-birth-weight infants. *NEJM*, **308**, 1330–1337.

CADMAN, D. & GOLDSMITH, C. (1986). Construction of social value or utility-based health indices: the usefulness of factorial experimental design plans. *J. Chron. Dis.*, **39**, 643–651.

CADMAN, D., GOLDSMITH, C. & BASHIM, P. (1984). Values, preferences and decisions in the care of children with developmental disabilities. *Develop. & Behav Pediat.*, **5**, 60–64.

CADMAN, D., GOLDSMITH, C., TORRANCE, G.W., BOYLE, M.H. & FURLONG, W. (1986). *Development of a Health Status Index for Ontario Children*. Final report to the Ontario Ministry of Health on Research Grant DM648 (00633). Hamilton: McMaster University.

CARTWRIGHT, A. (1957). The effect of obtaining information from different informants on a family morbidity inquiry. *App. Statist.*, **6**, 18–25.

CHESSELLS, J.M., NINANE, J. & TIEDEMANN, K. (1981). Present problems in management of childhood lymphoblastic leukaemia. Experience from the Hospital for Sick Children, London. In *Modern Trends in Human Leukaemia*. Neth, R., Gall, R.C., Graff, T., Mannweiler, R. (eds) pp. 108–114. Springer-Verlag: Berlin.

CLARRIDGE, B.R. & MASSAGLI, M.P. (1989). The use of female spouse proxies in common symptom reporting. *Med. Care*, **27**, 352–366.

CLAYTON, P.E., SHALET, S.M. & MORRIS-JONES, P.H. & PRICE, D.A. (1988). Growth in children tested for acute lymphoblastic leukemia. *Lancet*, **i**, 460–462.

FEENY, D., FURLONG, W., BARR, R.D., TORRANCE, G.W., ROSENBAUM, P. & WEITZMAN, S. (1992). A comprehensive multi-attribute system for classifying the health status of survivors of childhood cancer. *J. Clin. Oncol.*, **10**, 923–928.

GAYNON, P.S. (1990). Primary treatment of childhood acute lymphoblastic leukemia of non T cell lineage (including infants) in Childhood Acute Lymphoblastic Leukemia – Part II. Treatment, Present and Future. Pochedly, C., Civin, C.I. (eds) *Hematol. Oncol. Clinics of North America*, **4**, 915–936.

GREEN, D.M., ZEVON, M.A. & HALL, B. (1991). Achievement of life goals by adult survivors of modern treatment for childhood cancer. *Cancer*, **67**, 206–213.

GRIFFIN, N.K. & WADSWORTH, J. (1980). Effect of treatment of malignant disease on growth in children. *Arch. Dis. Child.*, **55**, 600–603.

HERJANIC, B. & REICH, W. (1982). Development of a structured psychiatric interview for children: agreement between child and parent on individual symptoms. *J. Abnorm. Child Psychol.*, **10**, 307–324.

KUPST, M.J. & SCHULMAN, J.L. (1988). Long-term coping with pediatric leukemia – a 6-year follow-up study. *J. Pediat. Psychol.*, **13**, 7–22.

KUPST, M.J., SCHULMAN, J.L., MAURER, H., HONIG, G., MORGAN, E. & FOCHTMAN, D. (1984). Coping with pediatric leukemia: a two-year follow-up. *J. Pediat. Psychol.*, **9**, 149–163.

LANSKY, S.B., LIST, M.A., LANSKY, L.L., RITTER-STERR, C. & MILLER, D.R. (1987). The measurement of performance in childhood cancer patients. *Cancer*, **60**, 1651–1656.

LEVINE, A.S. & HERSH, S.P. (1982). The psychological concomitants of cancer in young patients. In *Cancer in the Young*. Levine, A.S. (ed) pp. 367–387. Masson Publishing USA: New York.

LINKS, P.S. & STOCKWELL, M.L. (1985). Obstacles in the prevention of psychological sequelae in survivors of childhood cancer. *Amer. J. Pediat. Hematol./Oncol.*, **7**, 132–140.

MAGAZINER, J., SIMONSICK, E.M., KASHNER, T.M. & HOBEL, J.R. (1988). Patient-proxy response comparability on measures of patient health status and functional status. *J. Clin. Epidemiol.*, **41**, 1065–1074.

MAGUIRE, G.P., LITTMAN, P., FERGUSSON, J. & MOSS, K. (1987). The psychological sequelae of childhood leukemia. *Recent Results in Cancer Res.*, **88**, 47–56.

MEADOWS, A.T., GORDON, J. & MASSARI, D.J., LITTMAN, P., FERGUSSON, J. & MOSS, K. (1981). Declines in IQ scores and cognitive dysfunction in children with acute lymphocytic leukemia treated with cranial irradiation. *Lancet*, **ii**, 1015–1018.

MEADOWS, A.T., KREJMAS, N.L. & BELASCO, J.B. (1980). The medical cost of cure: sequelae in survivors of childhood cancer. In *Status of the Curability of Childhood Cancers*, van Eys, J. & Sullivan, M.P. (eds) pp. 263–276. Raven Press: New York.

MOSTOW, E.N., BYRNE, J., CONNELLY, R.R. & MULVIHILL, J.J. (1991). Quality of life in long-term survivors of CNS tumors of childhood and adolescence. *J. Clin. Oncol.*, **9**, 592–599.

NESBIT, M.E., KRIVIT, W., ROBISON, L. & HAMMOND, D. (1979). A follow-up report of long-term survivors of childhood acute lymphoblastic or undifferentiated leukemia. *J. Pediat.*, **95**, 727–730.

O'MALLEY, J.E., KOOCHER, G., FOSTER, D. & SLAVIN, L. (1979). Psychiatric sequelae of surviving childhood cancer. *Amer. J. Orthopsychiatry*, **49**, 608–616.

PASTORE, G., ZURLO, M.G. & ACQUAVIVA, A., CALCULLI, G., CASTELLO, M., CECI, A., DI TULLIO, M.L., GANDUS, S., MACCHIA, P., CORDERO DI MONTEZMOLO, L., MANDELLI, F., MASSOLO, F., NESPOLI, L., PAOLUCCI, G., ROSATE, M., SENESI, E., TAMARO, P., TRIPOLI, U. & TERRACINI, B. (1987). Health status of young children with cancer following discontinuation of therapy. *Med. & Pediat. Oncol.*, **15**, 1–6.

ROSENBAUM, P., CADMAN, D. & KIRPALANI, H. (1990). Pediatrics: assessing quality of life. In *Quality of Life Assessment in Clinical Trials*. Spilker, B. (ed) pp. 205–215. Raven Press: New York.

ROTHAM, M.L., HEDRICK, S.C., BULCROFT, K.A., HICKMAN, D.H. & RUBENSTEIN, L. (1991). The validity of proxy-generated scores as measures of patient health status. *Med. Care*, **29**, 115–124.

STATISTICS CANADA. (1992). *The 1991 General Social Survey* – Cycle 6, Health, Public Use Microdata File Documentation and Users' Guide. Statistics Canada: Ottawa.

TORRANCE, G.W., BOYLE, M.H. & HORWOOD, S.P. (1982). Application of multi-attribute utility theory to measure social preferences for health states. *Operations Res.*, **30**, 1043–1069.

TORRANCE, G.W., ZHANG, Y., FEENY, D., FURLONG, W.J. & BARR, R.D. (1992). Multi-attribute preference functions for a comprehensive health status classification system. McMaster University Centre for Health Economics and Policy Analysis Working Paper 92–18.

WHEELER, K., LEIPER, A.D., JANNOUN, L. & CHESSELLS, J.M. (1988). Medical cost of curing childhood acute lymphoblastic leukemia. *Br. Med. J.*, **296**, 162–166.

WHITT, J.K., WELLS, R.J., LAURIA, M.M., WILHELM, C.L. & MCMILLAN, C.W. (1984). Cranial radiation in childhood acute lymphoblastic leukemia. Neuropsychologic sequelae. *Amer. J. Dis. Childhood*, **138**, 730–736.